# Chemical Sensors Based on Cyclodextrin Derivatives

**DOI:** 10.3390/s8084961

**Published:** 2008-08-25

**Authors:** Tomoki Ogoshi, Akira Harada

**Affiliations:** 1 Department of Macromolecular Science, Osaka University / 1-1 Machikaneyama-cho, Toyonaka, Osaka 560-0043, Japan; 2 Department of Chemistry and Chemical Engineering, Graduate School of Natural Science and Technology, Kanazawa University / Kakuma-machi, Kanazawa, 920-1192, Japan

**Keywords:** Cyclodextrins, Chemical Sensors, Supramolecules, π-Conjugated Polymer, Nanocarbons, Nanoparticles

## Abstract

This review focuses on chemical sensors based on cyclodextrin (CD) derivatives. This has been a field of classical interest, and is now of current interest for numerous scientists. First, typical chemical sensors using chromophore appended CDs are mentioned. Various “turn-off” and “turn-on” fluorescent chemical sensors, in which fluorescence intensity was decreased or increased by complexation with guest molecules, respectively, were synthesized. Dye modified CDs and photoactive metal ion-ligand complex appended CDs, metallocyclodextrins, were also applied for chemical sensors. Furthermore, recent novel approaches to chemical sensing systems using supramolecular structures such as CD dimers, trimers and cooperative binding systems of CDs with the other macrocycle [2]rotaxane and supramolecular polymers consisting of CD units are mentioned. New chemical sensors using hybrids of CDs with π-conjugated polymers, peptides, DNA, nanocarbons and nanoparticles are also described in this review.

## Introduction

**Figure 1.**
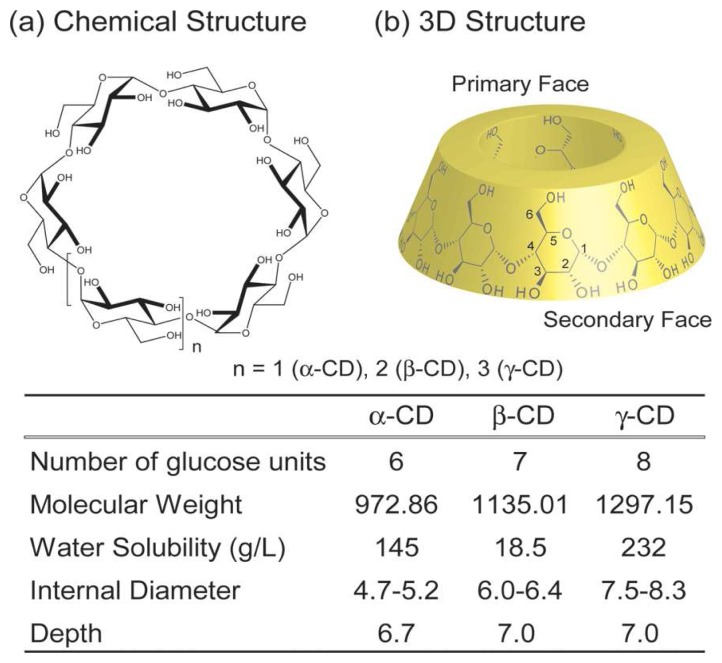
Structures of cyclodextrins (CDs).

Macrocyclic hosts such as crown ethers, cryptands, cyclophanes, calixarenes and cucurbiturils have been synthesized and received much attention, opening a wide range of opportunities for new supramolecular chemistry and materials. Among them, cyclodextrins (CDs) are the most important and promising macrocyclic hosts because they are water-soluble natural products, inexpensive, commercially available, nontoxic and readily functionalized [1-4]. CDs are cyclic oligosaccharides consisting of six or more glucopyranose units attached by *α*-1,4-linkages (Figure 1).

The most common CDs comprise six, seven and eight glucopyranose units and are named *α*-, *β*- and *γ*-CD, respectively. CDs form bucket structures, thus they display a large and narrow entrance. The narrow cavity side of CDs has primary hydroxyl groups and is called the primary face. In contrast, the large cavity side of CDs bears secondary hydroxyl groups and is called the secondary face. The difference between the primary and secondary hydroxyl groups allows selective functionalization on the primary and secondary rims. Well-established synthesis protocols for selective modification of CDs have been extensively studied by many researchers and have accelerated CD-related research [5-7]. One of the most important properties of CDs is the inclusion of guest molecules into their cavities. Because the hydroxyl groups of CDs on both faces arrange on the outside of the cavity and the inside of the CD cavity is a hydrophobic microenvironment, CDs can capture suitable hydrophobic guests into this hydrophobic cavity in aqueous media. The cavity size of CDs increases with the increasing number of glucopyranose repeating units. The cavity diameter of CDs is about 0.44 – 0.83 nm (*α*-CD: 0.49 nm, *β*-CD: 0.62 nm, *γ*-CD: 0.80 nm). Therefore, the cavities of *α*- and *β*-CDs are a suitable size for benzene rings and naphthalene molecules, respectively. Since the cavity size of *γ*-CD is larger thanthat of *α*- and *β*-CD, *γ*-CD captures large guests like fullerene and two aromatic guests in the cavity [8,9]. By employing the host-guest property, CDs have been applied for various fields such as biochemistry, material chemistry, catalysis and electronics. Especially, recent developments in supramolecular chemistry using CDs as a building blocks are extremely remarkable. Various supramolecular structures such as catenanes [10-12], rotaxanes [13-17], polyrotaxanes [18-20] and supramolecular polymers [21-23] have been reported.

The field of chemical sensors has been a growing research area and a wide range of books and reviews has been published in this field over the last three decades [24-29]. Among them, optical chemical sensors are quite interesting and useful because optical changes such as color and fluorescence by recognition of guest molecules can be directly and immediately seen with the naked human eye. Classically, chromophores, which exhibited optical changes with polarity variation of microenvironments, were employed as output signals. With marked advancement in the fields of polymer chemistry, biochemistry and nanotechnology, recent developments of new chemical sensors using π-conjugated polymers [24, 25], nanocarbons [26, 27] and nanoparticles [28, 29] have been significant.

In this review, we focus on chemical sensors using CD derivatives. In addition to classical chemical sensors consisting of chromophore appended CDs, new sensing systems based on metallocyclodextrins, supramolecular systems using CD dimers, trimers, hetero-host conjugates, rotaxane, supramolecular polymer, hybrids of organic polymers, nanocarbons and nanoparticles with CDs are described.

## Chemical Sensors Using Chromophore Appended CDs

The microenvironment of chromophores generally results in their color and fluorescence properties. Therefore, by introducing dye and fluorescence compounds into CDs, inclusion of guest molecules into the CD cavity can be detected. Chromophore-modified CDs have been studied for a long time by many researchers and a huge number of these sensors have been reported. Among them, Ueno and coworkers have pioneered the field of chromophore appended CD chemical sensors.

**Figure 2.**
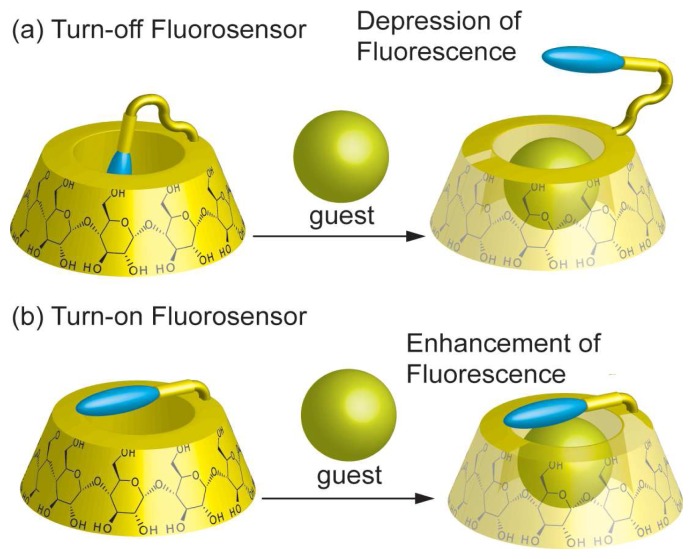
(a) Turn-off and (b) Turn-on fluorosensors.

They synthesized many kinds of “turn-off” fluorescent chemical sensors, in which fluorescence intensity was decreased by complexation with guest molecules (Figure 2a). In aqueous media, fluorophore-CD conjugates *via* a flexible linker forming a self-inclusion complex. When a competitive guest was added to the aqueous solution, the fluorophore was excluded from the inside to the outside of the CD cavity. The fluorescent CD exhibited strong emission in the self-inclusion state due to the hydrophobic environment of the CD cavity and exclusion of the fluorophore from the cavity to bulk aqueous media weakened its fluorescence intensity. They synthesized “turn-off” fluorescent chemical sensors of CD derivatives carrying fluorophore compounds such as dansyl [30-35], dimethylaminobenzoyl [36-38], naphthyl [39-41], pyrene [42] and anthracene [43,44] moieties. Moreover, a new type of “turn-on” fluorescent chemical sensors, in which fluorescence intensity was increased by formation of host-guest complexes, was also reported (Figure 2b). With a rigid spacer between CD and fluorophore, the fluorophore-CD conjugates could not form self-inclusion complexes. Therefore, the fluorophore moiety is surrounded by a hydrophilic environment, which resulted in suppression of the fluorescence. By inclusion of a hydrophobic guest molecule into the cavity of the fluorophore-CD conjugate, the fluorophore is located in a more hydrophobic environment, thus the fluorescence intensity increases. “Turn-on” type fluorescent chemical sensors such as 4-amino-7-nitrobenz-2-oxa-1,3-diazole-CD [45], napthol-CD [46] and hydroxyquinoline-CD [46] conjugates were reported.

**Figure 3.**
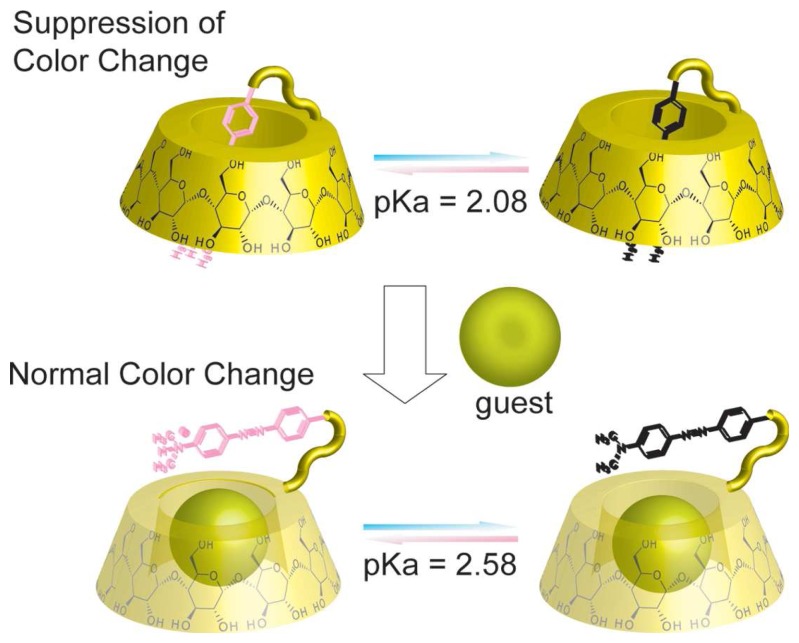
*p*-Methyl red appended *β*-CD chemical sensor.

Various kinds of dye moiety appended CD chemical sensors were also reported by Ueno and coworkers. Because pH indicators such as methyl red, *p*-nitrophenol and phenolphthalein show color changes depending on the pH conditions, they synthesized dye appended CD derivatives. In aqueous media, the dye moiety was included in the hydrophobic CD cavity (self-inclusion complex) and isolated from aqueous media (Figure 3).

Therefore, color changes from protonation/deprotonation of the dye with pH changes are suppressed. In contrast, upon addition of competitive guest molecules, the dye moiety was excluded from the CD cavity and located in the aqueous media. In that state, by environmental changes around the dye moiety, the dye moiety shows normal its color changes resulting from pH changes. Color change chemical sensors of CD derivatives carrying dyes such as methyl red [47,48], *p*-nitrophenol [49], alizarin yellow [50] and phenolphthalein [51] were reported.

## Chemical Sensors Using Metallocyclodextrins

**Figure 4.**
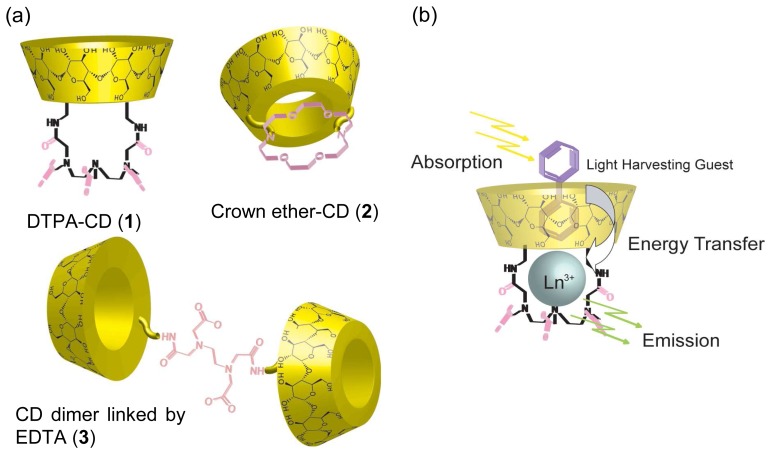
(a) Structures of DTPA (**1**), crown ether (**2**) and EDTA (**3**) appended CDs and (b) mechanism of absorption-energy transfer emission (AETE) process

Photoactive metal ion-ligand appended CDs, metallocyclodextrins [52], have been studied and applied as chemical sensors. By introducing metal binding sites such as diethylenetriaminepentaacetate (DTPA), crown ether and ethylenediaminetetraacetate (EDTA) moieties into CDs, fluorescent sensors have been constructed. One of the major sensing mechanisms using metallocyclodextrins is an absorption-energy transfer emission (AETE) process, which involves excitation of the light harvesting guest, energy transfer to a metal ion and subsequent luminescence from the metal ion. Lanthanide ions such as Eu(II) and Tb(II) are mainly used because of their long-lifetimes and strong fluorescence. The complexes of lanthanide ion with binding site modified CDs such as DTPA-CD (**1**, Figure 4a) [53] and crown ether-CD conjugates (**2**, Figure 4a) [54] showed little fluorescence in aqueous media because of a lack of aromatic hydrocarbons (light harvesting groups).

By inclusion of aromatic guests such as toluene, benzene and biphenyl into their CD cavity, strong fluorescence from the lanthanide was observed (Figure 4b). These aromatic guests act as light harvesting groups and energy transfer from these guests to the lanthanide occurred. After excitation of the lanthanide by the energy transfer, luminescence from the lanthanide took place. These fluorosensors are assigned to “turn-on” chemical sensors because the fluorescence is increased by molecular recognition events.

Reinhoudt and coworkers synthesized an EDTA-linked *β*-CD dimer (**3**, Figure 4a) [55]. The complex between **3** and lanthanides also showed AETE upon addition of adamantane dimer linked by a biphenyl group. Inclusion of both adamantyl ends into the *β*-CD cavities of **3** resulted in AETE from the biphenyl group to the lanthanide. Lanthanide complexes with polypyridine [56] and heptabipyridine [57] modified CD also showed AETE properties and acted as chemical sensors towards guest molecules.

**Figure 5.**
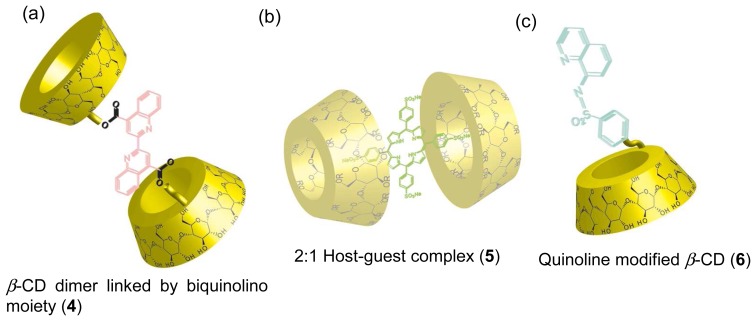
Structures of (a) *β*-CD dimer linked by biquinolino group (**4**), (b) 2:1 host-guest complex of alkylated *β*-CD and *meso*-tetraphenylporphyrin (**5**) and (c) *β*-CD having 6-methoxy-(8-*p*-toluenesulfonamido)quinoline (**6**).

Transition metal ion-ligand appended CDs have been also studied. *β*-CD dimer conjugated by biqunolino group (**4**, Figure 5a) formed complex with Cu(II) ion [58]. Complex between **4** and Cu(II) acted as efficient fluorescence sensors and showed remarkable fluorescence enhancement by formation of a 1:1 sandwich inclusion complex with a steroid guest.

A 2:1 host-guest complex of alkylated *β*-CD and *meso*-tetraphenylporphyrin (**5**, Figure 5b) was used as a selective and sensitive fluorescence system for Zn(II) [59]. Due to enhancement of the porphyrin fluorescence by formation of metalloporphyrin, the complex, **5**, is applied to sense and detect metal ions in water. The complex showed excellent selectivity for Zn(II) over the several metal ions. *β*-CD having 6-methoxy-(8-*p*-toluenesulfonamido)quinoline (**6**, Figure 5c), which exhibited high selectivity for Zn(II) as compared to Ca(II), Mg(II) and other metal ions, was also synthesized. Compound **6** showed high sensing ability for Zn(II) and will be expected to be useful as imaging reagent of Zn(II) in living tissue or in cells [60].

## Chemical Sensors Using CD Supramolecular Systems

Supramolecular structures constructed of CD moieties as building blocks are quite interesting and have attracted tremendous interest. One of the applications of supramolecular structures is the construction of chemical sensors by using cooperative recognition systems and dynamic changes of supramolecular structures toward chemicals and ions.

**Figure 6.**
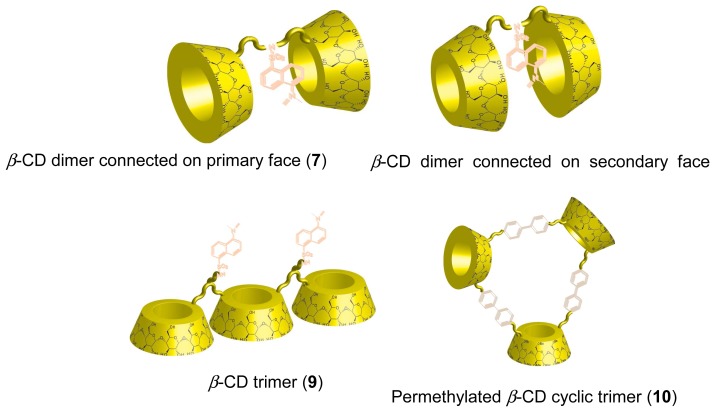
Fluorescent CD dimers and trimers

Covalently coupled CDs such as CD dimers and trimers form large hydrophobic binding cavities, thus they strongly capture large guest compounds compared to monomeric CDs. Therefore, sensing systems based on CD dimers and trimers have been investigated. Ueno *et al.* first synthesized dansyl modified *β*-CD dimer (**7**, Figure 6) [61]. **7** exhibited remarkable molecular recognition for steroid compounds, which was different from native *β*-CD. The phenomenon results form large hydrophobic binding site of the *β*-CD dimer structure. By inclusion of the steroid guests into the hydrophobic space between *β*-CDs, fluorescence from the dansyl moiety was diminished. This is because the dansyl moiety included into the cavity of *β*-CD dimer is excluded from the hydrophobic environment into the hydrophilic aqueous media. Reinhoudt and coworkers prepared a fluorescent *β*-CD dimer, in which *β*-CDs were connected on their secondary face (**8**, Figure 6) [62]. The host-guest properties of the *β*-CD dimer connected on the secondary face is different from that of a *β*-CD dimer connected on the primary side. Therefore, **8** exhibited different fluorescence changes upon addition of guest molecules. A fluorescent *β*-CD trimer having two dansyl groups at linkers between CDs, (**9**, Figure 6) was also synthesized. In this system, **9** effectively captured guest molecules [63]. Kuroda and coworkers synthesized new permethylated *β*-CD dimer and cyclic trimer (**10**, Figure 6) bridged with biphenyl moieties. An anthracene derivative having two hydrophobic alkyl chains was strongly captured by the cyclic permethylated *β*-CD trimer (**10**) [64].

**Figure 7.**
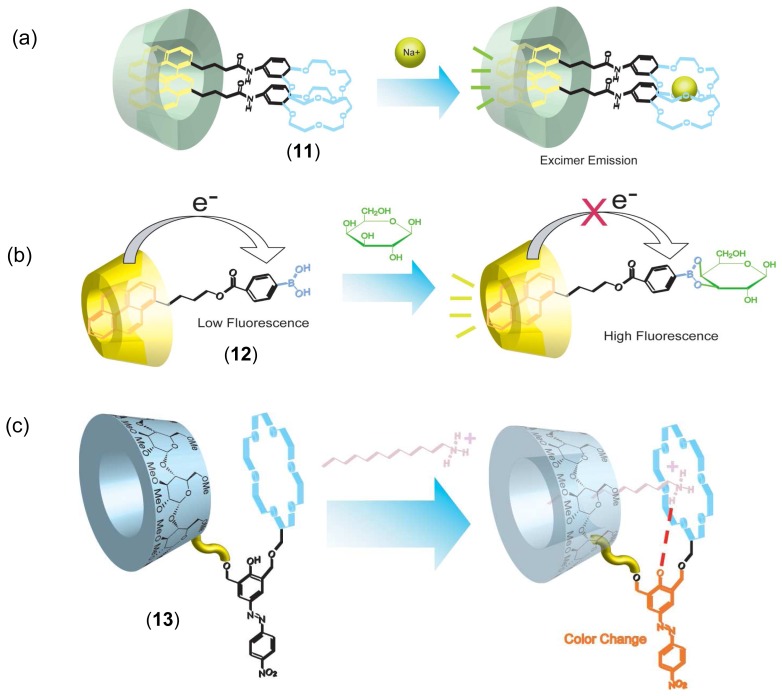
Cooperative binding systems of hybrids of (a) *γ*-CD with pyrene-crown ether (**11**), (b) *β*-CD with pyrene-boronic acid (**12**) and (c) methylated *α*-CD-crown ether-azophenyl dye (**13**) conjugates.

Cooperative binding systems using hybrids of CD with the other macrocyclic hosts were also reported. Hayashita and coworkers constructed a high selective molecular recognition system based on host-guest complexes between *γ*-CD and pyrene-crown ether conjugates [65,66]. In the presence of *γ*-CD, pyrene-crown ether conjugate and K(I) ion, pyrene monomer emission disappeared and excimer emission appeared due to formation of 2:1 sandwich type dimer between crown ether and K(I) ion (**11**, Figure 7a). A boronic acid fluorophore/*β*-CD complex chemical sensor for selective sugar recognition was also reported (**12**, Figure 7b) [67].

Cyclodextrin-crown ether conjugates were also used for chemical sensors. A CD-crown ether-azophenyl dye conjugate (**13**, Figure 7c) was synthesized by Kaneda *et al.* [68, 69]. Upon addition of primary and secondary alkyl amines, **13** showed color changes in aqueous media. In contrast, no color change was observed upon addition of tertiary alkyl amines to the aqueous solution. The main reasons to the selectivity of **13** toward amines are cooperative binding of both the crown ether and CD moieties.

The crown ether strongly binds primary and secondary ammonium ions and the CD forms a complex with the lipophilic alkyl tail of the amine. Therefore, the primary and secondary alkyl amines form strong complexes with **13**. Reinhoudt and coworkers prepared CD-calix[4]arene conjugates [70]. The fluorophore was attached to the upper rim of calix[4]arene moiety. The fluorophore was included intra-molecularly into the CD cavity in aqueous media. Upon addition of guests such as steroids and terpenes, fluorescence intensity decreased (turn-off type fluorescent chemical sensor), indicating exclusion of the fluorophore from CD cavity to aqueous solution. Its complexation properties were different from those of native CDs, since the appended calix[4]arene moiety expanded the hydrophobic microenvironment. Chemical sensors using cooperative multiple recognition by calix[4]arene bridged *β*-CD dimer were also reported [71].

**Figure 8.**
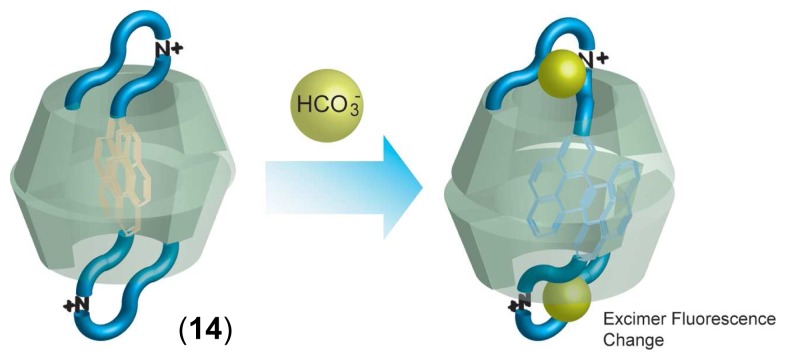
Chemical structure of pyrene appended *γ*-CD *via* triamine spacer (**14**) and proposed sensing mechanism for HCO_3_^-^ anion

Suzuki and coworkers reported interesting chemical sensors using a supramolecular double-threaded *γ*-CD dimer (Figure 8). They synthesized pyrene appended a *γ*-CD *via* a triamine spacer (**14**, Figure 8) [72]. In aqueous solution (pH = 7-9) **14** showed strong excimer fluorescence, indicating that **14** formed supramolecular double-threaded *γ*-CD dimers in aqueous media. The excimer emission from the *γ*-CD dimers was changed by adding only HCO_3_^-^ anion, while the other anions did not cause changes in excimer emission. These observations indicate high selectivity for HCO_3_^-^ anion. In neutral condition, triamine spacer forms *peudo*-azacrown ring with one charged ammonium group and two amino groups. The *peudo*-azacrown ring selectively binds HCO_3_^-^ anion, leading to the change in excimer emission.

**Figure 9.**
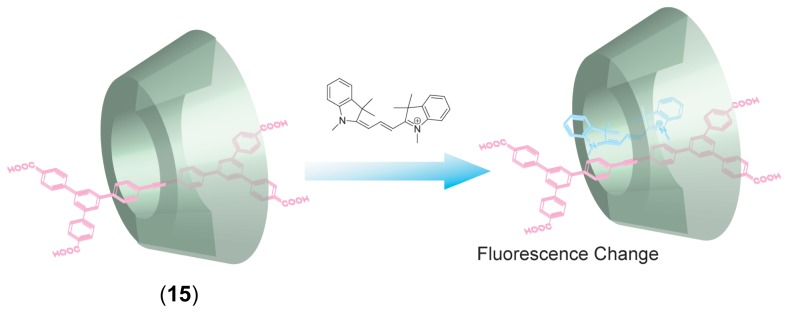
Chemical structure of [2]rotaxane with stilbene as axle, terphenylenedicarboxylic acid as bulky stopper and *γ*-CD as ring (**15**) and inclusion of guests on hydrophobic floor of stilbene.

Anderson and coworkers reported unique chemical sensor using [2]rotaxane [73]. A [2]rotaxane with stilbene as axle, terphenylenedicarboxylic acid as bulky stopper and *γ*-CD as ring, (**15**, Figure 9) has a substantial cavity, thus suitable for another guest to be included into the remaining space. The stilbene in *γ*-CD acts as hydrophobic floor to the cavity of the *γ*-CD, leading to a 1,000-fold increase in its affinity for suitable guests. The stilbene also plays a role as a fluorophore in order to report formation of the host-guest complex between substantial cavity of the [2]rotaxane and guests.

Chemically responsive supramolecular polymers have been reported by our group, [74,75]. It was well known that guest moiety appended CDs with rigid spacers formed supramolecular polymers. Among them, a [*N*-(2,4,6-trinitrophenyl)-6-amino-*trans*-cinnamoyl]-*β*-CD appended CD derivative formed a supramolecular hydrogel based on host-guest and hydrogen bond interactions. The hydrogel was chemically responsive and changed to sol state upon addition of competitive guest, host and denaturing compounds.

## Chemical Sensors Using Polymers Carrying CDs

**Figure 10.**
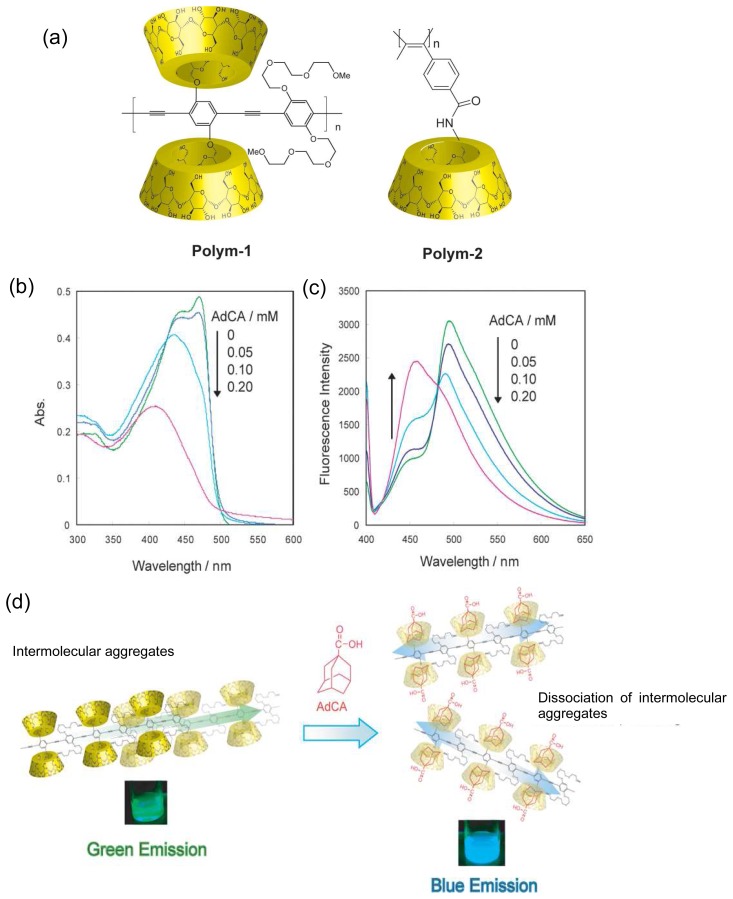
(a) Chemical structures of **Polym-1** and **Polym-2**. (b) UV-Vis and (c) emission spectral changes (excited at 400 nm) of aqueous **Polym-1** solutions (0.020 mM) by adding AdCA. (d) Proposed mechanism of dynamic structural changes upon addition of AdCA.

π-Conjugated polymers are attracting significant interest because they are used as electrical conductivity, electroluminescence, light-emitting diodes and chemical sensors. Among them, one of the current interests in the field of conjugated polymers focuses on tuning of their optical and electrical properties by stimulus such as oxidation-reduction, pH and metal cation. We synthesized water-soluble chemically-responsive fluorescent polymer by employing CD. *β*-CD modified poly(phenylene ethynylene) (**Polym-1**, Figure 10a) was synthesized [76]. **Polym-1** is structurally interesting wherein a poly(phenylene ethynylene) is flanked by two *β*-CD groups. **Polym-1** showed blue fluorescence in *N*,*N*-dimethylformamide (DMF), while green fluorescence was observed in aqueous media. In aqueous solution, **Polym-1** is not completely soluble in monomeric form but forms partially quenched aggregates of poly(phenylene ethynylene) backbone. Fluorescence of **Polym-1** was changed by addition of guest compounds. Fluorescence color change and quenching of **Polym-1** were induced by aliphatic and acceptor guests, respectively. With aliphatic 1-adamantanecarboxylic acid (AdCA) as a guest compound, UV-Vis absorption peak at 470 nm derived from the stacking between π-conjugated polymer backbones largely decreased and completely disappeared with 0.20 mM of AdCA (Figure 10b). The emission with the peak at 490 nm resulted from the intermolecular π-stacking between polymer chains also decreased and a new emission peak around 460 nm appeared with an increase in the concentration of AdCA (Figure 10c).

The emission color was changed from light green (fluorescence quantum yield: **Φ**_fl_ = 0.07) to deep blue (**Φ**_fl_ = 0.11) by addition of AdCA (Figure 10d). These observations indicate dissociation of intermolecular π-stacking of polymer backbones by complexation of AdCA to the *β*-CD moieties of **Polym-1**. Repulsive forces between the complexed anions might prevent polymer chains from aggregating.

**Figure 11.**
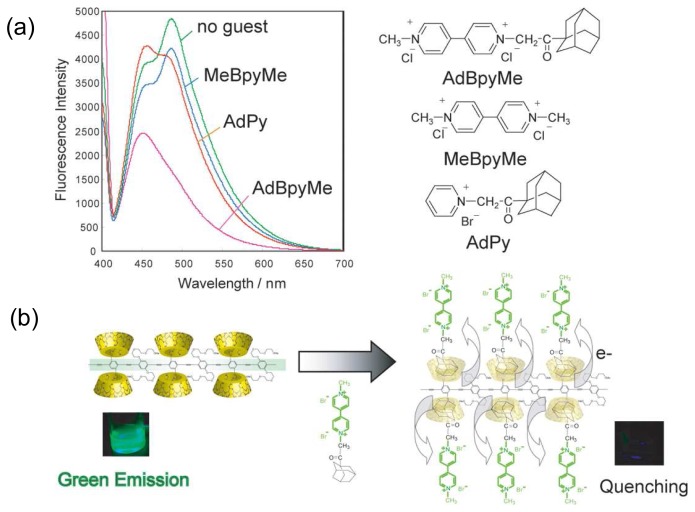
(a) Emission spectral changes (excited at 400 nm) of aqueous **Polym-1** solutions (0.020 mM) by adding viologen derivatives (0.20 mM). (b) Proposed mechanism of electron transfer from polymer backbone to viologen group.

Figure 11 shows changes in fluorescence by adding viologen derivatives. The fluorescence from poly(phenylene ethynylene)s was quenched by electron accepting viologen groups. By adding adamantane modified viologen group (AdBpyMe), large fluorescence quenching was observed (Figure 11a). On the other hand, by using *N*,*N*^′^-dimethyl-4,4′-bipyridinium (MeBpyMe), low fluorescence quenching occurred because the cationic viologen group barely formed a host-guest complex with CD. These data indicate that the fluorescence quenching by adding AdBpyMe is due to formation of the inclusion complex between adamantane moiety of AdBpyMe and *β*-CD of **Polym-1**. Holding viologen groups on the side chain of **Polym-1** *via* host-guest formation results in efficient electron transfer from the poly(phenylene ethynylene) backbone to the viologen moiety of AdBpyMe (Figure 11b). Upon addition of adamantane carrying pyridinium moiety (AdPy) instead of AdBpyMe, the fluorescence quenching was not observed because the pyridinium group did not act as an efficient electron acceptor of poly(phenylene ethynylene)s. The chemically responsive fluorescence color change and quenching system in aqueous solution were interesting and little known.

Yashima and coworkers synthesized helical polyacetylene-carrying CD moieties (**Polym-2**, Figure 10a) [77-79]. **Polym-2** showed a visible color change with a dynamic change in the helical pitch of the conjugated backbone induced by inclusion of guest molecules into CD cavity. Moreover, helicity of **Polym-2** can be also switched by complexation with chiral guests. With (*S*)-1-phenylethylamine, a color change from yellow to red was observed. In contrast, no color change was found upon addition of (*R*)-1-phenylethylamine.

**Figure 12.**
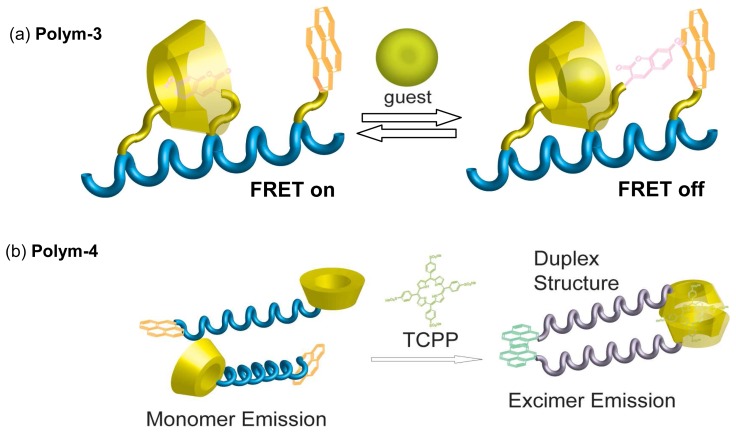
Schematic representation for the guest-induced structural change of (a) peptide-CD-chromophore (**Polym-3**) and (b) DNA-CD-chromophore (**Polym-4**) conjugates.

Peptides are designable polymers by using solid phase synthesis, thus construction of sensing systems based on peptide-CD conjugates has been studied by Ueno and coworkers. They designed various peptides having CD and chromophore moieties. For example, they synthesized a CD-peptides conjugate, (**Polym-3**, Figure 12a) bearing a pyrene donor and a coumarin acceptor in the side chains [80, 81]. In aqueous media, **Polym-3** showed strong fluorescence from the coumarin group by excitation at the absorption wavelength of pyrene. The observation indicates that intramolecular fluorescence resonance energy transfer (FRET) from pyrene to coumarin takes place. The coumarin moiety is intramolecularly included into a hydrophobic *β*-CD cavity, therefore the fluorescence from the coumarin unit is not suppressed. Upon addition of a competitive guest molecule, the fluorescence from the coumarin moiety decreased. The observation derives from exclusion of the coumarin group from inside to outside of the *β*-CD cavity and association between the coumarin and the pyrene units appended peptide backbone. CD-peptide conjugates with naphthyl [82], ionophore and dansyl [83], pyrene and nitrobenzene [84] moieties were also constructed.

Chemical sensor based on fluorophore-DNA-methylated *β*-CD conjugate (**Polym-4**, Figure 12b) has been studied recently [85]. When the methylated *β*-CD moiety of **Polym-4** captured TPPS in a 2:1 binding manner, duplex structure of DNA was induced. By formation of the duplex structure, emission switching from pyrene monomer to excimer occurred.

## Chemical Sensors Using Cyclodextrin-Nanocarbon Hybrids

**Figure 13.**
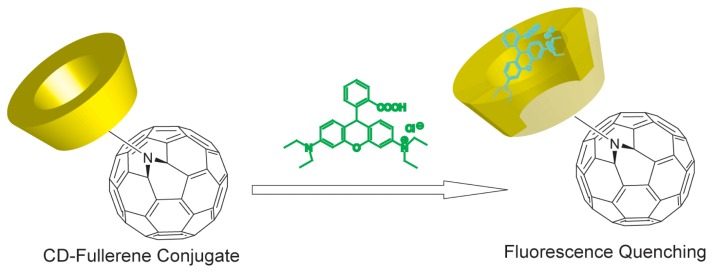
Chemical structure of fullerene appended CD and fluorescence quenching by inclusion of Rhodamine B.

Nanoscale carbon materials such as fullerene, carbon nanotubes and nanodiamonds have attracted great attention because of their unique structural, electrical, and mechanical properties. One of the recent new challenges for construction of CD based chemical sensors is hybridization of CD with nanocarbon materials. Yuan and Fujita *et al.* first reported the synthesis of defined structure of fullerene-CD conjugates *via* 1.3-dipolar cycloaddition of CD azides to fullerene (Figure 13) [86]. The fullerene-CD hybrids captured fluorescent guest, Rhodamine B and quenched the fluorescence.

**Figure 14.**
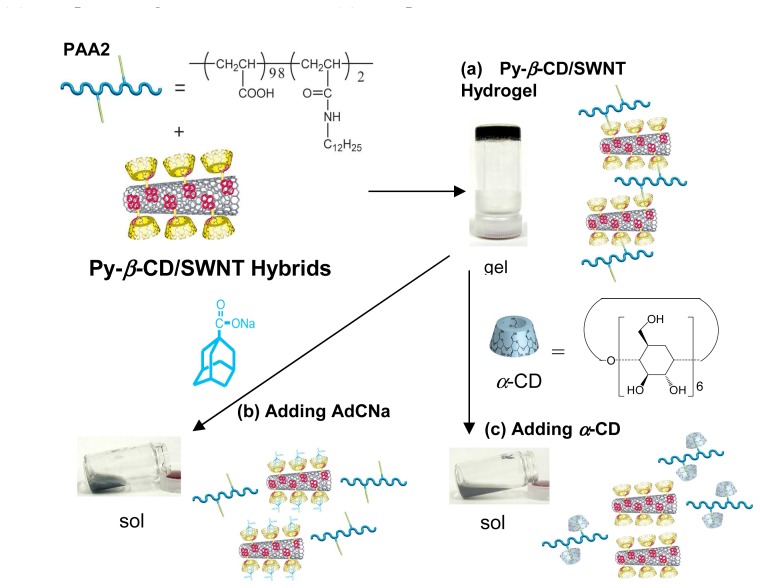
(a) Py-*β*-CD/SWNT hydrogel with **PAA2**. Gel to sol transitions upon addition of (b) competitive guest, AdCNa and (c) competitive host, *α*-CD.

We recently reported the synthesis of carbon nanotube-CD hybrids and construction of novel chemically-responsive supramolecular SWNT hydrogel systems [87]. Because CDs show high solubility in water, water-soluble SWNTs carrying CDs are obtained by using physical adsorption of pyrene modified *β*-CDs (**Py-*β*-CDs**) on SWNT surfaces (**Py-*β*-CD/SWNT hybrids**, Figure 14). Since vacant CD cavities of **Py-*β*-CDs** around SWNT are able to capture guest molecules on SWNT surface, supramolecular SWNT hydrogels are prepared by utilizing host-guest interaction between the *β*-CDs of **Py-*β*-CD/SWNT hybrids** and polymers carrying guest moieties. Supramolecular SWNT hydrogels were formed by host-guest interactions between the *β*-CDs of **Py-*β*-CD/SWNT hybrids** and dodecyl groups (2 mol%) modified poly(acrylic acid) (**PAA2**, Figure 14a). Furthermore, SWNT hydrogels composed of **Py-*β*-CD/SWNT hybrids** and **PAA2** changed to sol by adding competitive guest or host compounds. When sodium adamantane carboxylate (AdCNa) was added to the hydrogel as a competitive guest, gel to sol transition was observed (Figure 14b). This result indicates dissociation of the host-guest complexes between the *β*-CDs of **Py-*β*-CD/SWNT hybrids** and dodecyl groups of **PAA2** because AdCNa strongly interacts with the *β*-CD compared with the dodecyl group. Upon addition of *α*-CD as a competitive host, the gel also changed to sol (Figure 14c). It is because dodecyl moieties form complexes more favorably with *α*-CD than with *β*-CD. To the best of our knowledge, it is the first example of construction of SWNT-CD hybrids and gel to sol changeable SWNT hydrogel *via* supramolecular formation. Stoddart and Grüner *et al.* reported application of the pyrene-CD decorated SWNT hybrid field-effect transistors (FETs) as chemical sensors to detect organic guests [88]. FET characteristics of SWNT-CD hybrids were highly sensitive and depended on association constants between organic molecules and CDs. As the association constants between CDs and guests increased, larger changes in the FET characteristics were observed. The proposed mechanisms are either the change of the carrier concentration as a result of the change in the charge transfer from pyrene modified cyclodextrin to the SWNTs or the change of carrier mobility resulting from deformation of SWNTs. CD-multiwalled carbon nanotubes (MWNTs) conjugate by reaction of surface-bond carboxylic chloride groups of MWNTs with amino CDs was reported [89]. By complexation with tetrakis(4-carboxyphenyl)porphyrin (TCPP), fluorescence quenching was observed due to the photoinduced electron transfer from TCPP to MWNTs.

## Chemical Sensors Using CD-Nanoparticle Hybrids

**Figure 15.**
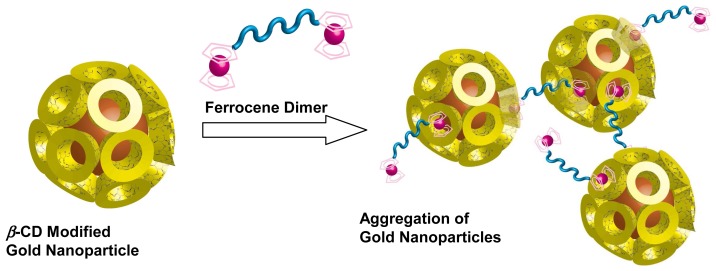
Formation of network aggregate of *β*-CD modified gold nanoparticles upon addition of ferrocene dimer

When gold nanoparticles aggregate, a color change from red to purple and blue is observed. This phenomenon can be promisingly applied to optical sensing devices. Kaifer and coworkers synthesized *β*-CD modified gold nanoparticles [90,91]. Addition of guest ferrocene dimer to colloidal dispersion of *β*-CD-modified gold nanoparticles initially caused a red shift and then precipitation of a red solid (Figure 15). In contrast, these red shift and precipitation were not observed upon addition of ferrocene-methanol. These data indicate that ferrocene dimer acts as a linker between different gold nanoparticles, leading to their aggregation. The color change resulting from aggregation of *β*-CD modified gold nanoparticles by adding ferrocene dimer will be applied for chemical sensor toward guest molecules. Furthermore, they also synthesized *γ*-CD modified gold nanoparticles. By mixing fullerene (C_60_) with colloidal solutions of *γ*-CD modified gold nanoparticles, network aggregates formed. The foundation results from formation of 2:1 host-guest complexes between *γ*-CD attached to different nanoparticles and C_60_ [92]. Formation of the network aggregate should be useful for sensing system of C_60_.

A new nanobiosensor for glucose based on FRET between concanavalin A (ConA) modified CdTe quantum dots and *β*-CD modified gold nanoparticles has been recently reported [93]. Quantum dots such as CdS, CdTe and ZnS nanoparticles show superior photoluminescence quantum yields and color changes depending on the size of their diameters. The sensing strategy is based on FRET between CdTe quantum dots as energy donor and gold nanoparticles as energy acceptor. When CdTe quantum dots form assembles with gold nanoparticles, the fluorescence from quantum dots is decreased by FRET effect. By mixing *β*-CD modified gold nanoparticles and ConA conjugated CdTe quantum dots, assemblies between these gold nanoparticles and CdTe quantum dots took place. Because ConA binds *β*-CD, FRET from CdTe quantum dots to gold nanoparticles occurs. By adding glucose to the hybrid system, fluorescence from CdTe quantum dots increased due to low FRET effect. It is because the glucose competes with *β*-CD on the binding site of ConA and the assemblies of *β*-CD modified gold nanoparticles and ConA conjugated CdTe quantum dots are dissociated.

## Conclusions and Outlook

The area of chemical sensors using CD derivatives is very important, although their history dates back less than 30 years. Classically, chromophore modified CD chemical sensors were constructed. However, with progress and improvements in other fields such as supramolecular chemistry, polymer chemistry, biochemistry, inorganic chemistry and nanotechnology, the research has focused on the construction of sophisticated sensing systems by hybridization with ideas from these other fields. Combinations of CD chemistry with the other chemistries and concepts have enabled elaboration of novel chemical sensors. New CD chemical sensors with high additive values such as high sensitivity, wide versatility and high functionality should be created with further progress of the other research fields.
